# Natural Products from Endophytic Fungi Associated with Rubiaceae Species

**DOI:** 10.3390/jof6030128

**Published:** 2020-08-07

**Authors:** Jacqueline Santos Cruz, Carla Amaral da Silva, Lidilhone Hamerski

**Affiliations:** 1 Department of Chemistry, Military Institute of Engineering, Praça General Tibúrcio 80, Rio de Janeiro 22290-270, Brazil; jacquelinescruz@hotmail.com; 2 Walter Mors Institute of Research on Natural Products, Federal University of Rio de Janeiro, Rua Carlos Chagas Filho 373, Rio de Janeiro 21941-902, Brazil; carlota.amaral@gmail.com

**Keywords:** Rubiaceae, endophytes, fungi, antibacterial, antifungal, neurodegenerative diseases, anti-inflammatory

## Abstract

This review presents the chemical diversity and pharmacological properties of secondary metabolites produced by endophytic fungi associated with various genera of Rubiaceae. Several classes of natural products are described for these endophytes, although, this study highlights the importance of some metabolites, which are involved in antifungal, antibacterial, anti-protozoal activities; neurodegenerative diseases; cytotoxic activity; anti-inflammatory and antioxidant activity; and hyperglycemic control.

## 1. Introduction

Natural products are small molecules from primary and secondary metabolites naturally synthesized by microorganisms, plants, or animals [1,2]. They are a continuing source of novel bioactive metabolites and have a significant impact on modern medicine [3,4]. Currently, more than 70% of antibacterial and anticancer compounds are natural products or their derivatives [5,6].

Fungi-derived natural products are considered one of the most relevant sources discovery and molecular diversity for new drugs. They are valuable source of biological metabolites that find wide-ranging applications as antibiotics, antifungal, immunosuppressants, antiparasitic and anticancer agents [7,8,9,10,11,12]. Among the microorganisms, endophytes have aroused interest in the last decades mainly for the discovery of important secondary metabolites identified from them.

The term endophyte refers to the microorganism that colonizes interior organs of plants, generally inhabiting their aerial parts such as stems and leaves, but that does not have pathogenic effects on its host [1,7,13,14,15,16]. Endophytes are ubiquitously found in every plant species examined to date. It is worth mentioning that, of the nearly 300,000 species on earth, each plant hosts one or more endophytes, and approximately 1 million of different species of microorganisms can be found [1,17]. In their symbiotic association, the host plant protects and feeds the endophyte, which in return produces bioactive metabolites to enhance the growth and competitiveness of the host and to protect it from herbivores and plant pathogens [7,9,18].

Endophytic fungi are known to produce a wide range of bioactive secondary metabolites, emphasizing chemical diversity, molecules originality and their biological activities [1,7,9,15,16,17,19,20,21,22,23,24]. Some studies suggest that up to 51% of bioactive metabolites obtained from endophytic fungi have unknown chemical structure, which highlights the huge biotechnological potential of this microbial group to the discovery of new drugs [21].

This review will focus on secondary metabolites synthesized by endophytic fungi isolated from Rubiaceae species, as well as the biological activities described in the literature for these compounds. The bibliographic research was carried out until March 2020.

## 2. Secondary Metabolites Produced by Endophytic Fungi from Rubiaceae

Rubiaceae is the fourth largest angiosperm family and comprises about 617 genera and 13,000 species of herbs, shrubs, and trees, found worldwide, especially in tropical and warm regions [25,26,27]. This family presents a vast diversity of chemical substances such as iridoids, anthraquinones, indole alkaloids, terpenoids, flavonoids, and alkaloids [28,29,30,31,32]. Diverse species of Rubiaceae have widespread use in folk medicine, and some of them showed anti-inflammatory, analgesic, antibacterial, mutagenic, antiviral, and antioxidant activities. Besides, an effect on vascular diseases and action on the central nervous system were observed [25,27,33,34]. 

The research on microorganisms associated with the Rubiaceae family, for biotechnological applications, led to the isolation of endophytic fungi [35,36,37,38,39,40,41,42,43,44,45,46,47] and the discovery of several bioactive metabolites [38,48,49,50,51,52,53,54,55]. The diversity of chemical structures observed for secondary metabolites synthesized by fungi isolated from Rubiaceae species showed a dynamic range of metabolites pathways used by these microorganisms.

**Figure 1:**
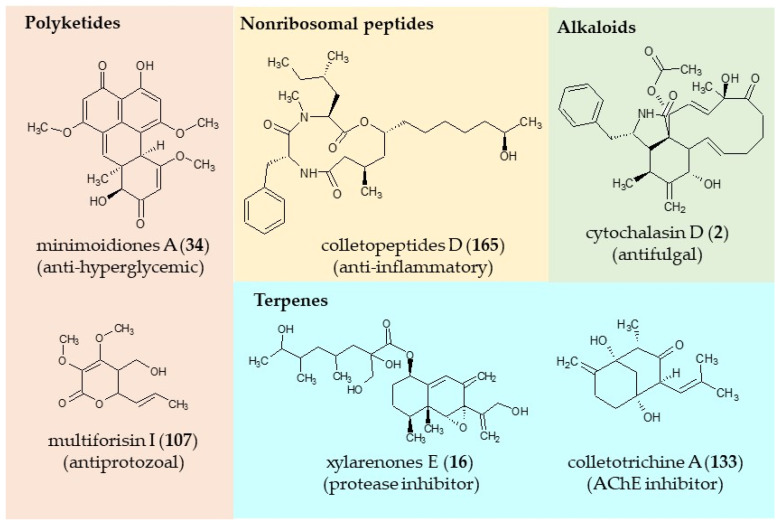
Examples of fungal natural products produced by endophytic fungi associated with various genera of Rubiaceae grouped by biosynthetic origin.

Fungi secondary metabolites are categorized in chemical classes: polyketides [56,57,58,59], non-ribosomal peptides [57,59,60,61,62], ribosomal peptides [62,63,64], terpenes [65,66,67], and hybrid metabolites [68,69,70,71,72,73,74]. These chemical classes are synthesized by specialized class-defining (backbone) enzymes such as polyketides synthases (PKSs), non-ribosomal peptide synthetases (NRPSs), terpene cyclases (TCs), and dimethylallyl tryptophan synthases (DMATSs), respectively. The endophytes isolated from Rubiaceae showed the ability to produce these chemical classes (Figure 1). The set of enzymes needed for the production of a secondary metabolite is encoded by a gene cluster (BGC). Interestingly the genes that are essential for the synthesis of a primary metabolite are dispersed throughout the fungal genome, while the genes encoding the enzymatic activities for metabolic pathways to produce any secondary metabolite are arranged in continuous fashion. In the last decades, significant advances have been observed in the identification, understanding, and engineering of fungal biosynthetic gene clusters (BGCs) [75,76,77,78,79,80,81,82,83,84].

**Figure 2:**
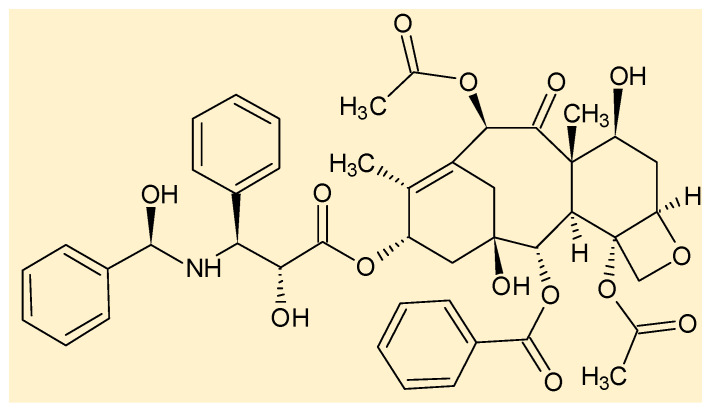
Taxol, the first compound isolated from an endophytic fungus from Rubiaceae.

The endophytic fungi distribution and diversity in Rubiaceae have been reported since the 1950s [85,86,87], and studies performed with *Coffea arabica* stand out [88,89,90,91,92]. However, only in 1999 was the first study on the secondary metabolism of endophytic fungi isolated from Rubiaceae species published. In this work, Strobel related the occurrence of taxol (Figure 2), a potent anticancer drug, in the culture of the endophyte *Seimatoantlerium tepuiense* (Amphisphaeriaceae) isolated from *Maguireothamnus speciosus* [93]. The occurrence of taxol was described for other endophytes isolated from Rubiaceae species, *Botryodiplodia theobromae* (Botryosphaeriaceae) and *Aspergillus oryzae* (Trichomaceae), obtained from *Morinda citrifolia* and *Tarenna asiatica*, respectively [94,95,96]. The pharmacological properties of taxol, isolated from *Botryodiplodia theobromae*, were confirmed through the cytotoxicity assay [94].

**Figure 3:**
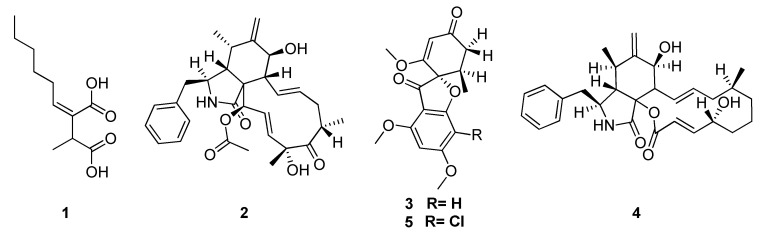
Five compounds extracted from *Xylaria* sp.

The secondary metabolites study on microorganisms associated with Rubiaceae continued with *Palicourea marcgravii* St. Hil. It was popularly known as “erva de rato” and provided several endophytic fungi, including a *Xylaria* sp. (Xylariaceae) isolated from their leaves. The crude extract from *Xylaria* sp. showed a potential antifungal activity, and five compounds: 2-hexyl-3-methyl-butanodioic acid (**1**), cytochalasin D (**2**), 7-dechlorogriseofulvin (**3**), cytochalasin B (**4**) and griseofulvin (**5**) were obtained (Figure 3) [97].

**Figure 4:**
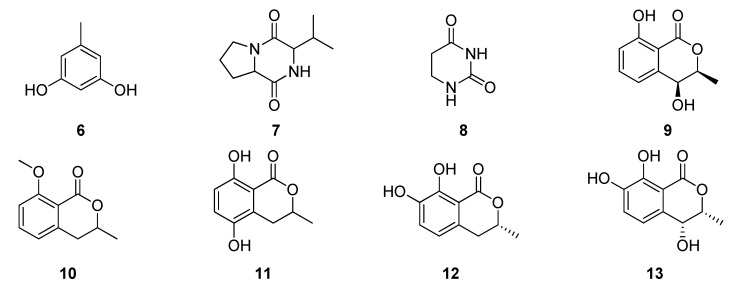
Compounds extracted from *Penicillium* species.

Oliveira et al. (2009) explored endophytic fungi living in plants of the Brazilian flora; two *Penicillium* (Trichocomaceae) species from leaves of *Alibertia macrophylla* were isolated. *Penicillium* sp.1 was cultivated in corn and potato dextrose broth produced three different compounds: orcinol (**6**), cyclo-(L-Pro-L-Val) (**7**), uracil (**8**). The acetonitrile fraction from *Penicillium* sp.2 led to three dihydroisocoumarins: 4-hydroxymellein (**9**), 8-methyl-mellein (**10**) and 5-hydroxymellein (**11**) [98]. Additionally, (R)-7-hydroxymellein (**12**) and (3R,4R)-4,7-dihydroxymellein (**13**) were also isolated from *Penicillium* sp. associated with *A. macrophylla* (Figure 4) [99].

**Figure 5:**
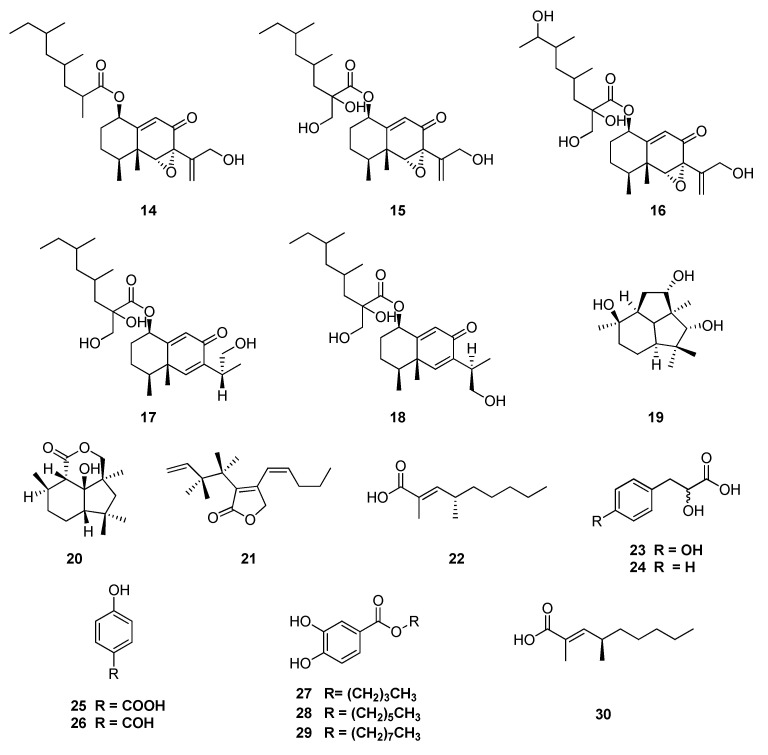
Compounds extracted from endophytes associated on *A. macrophylla.*

The continuing search for endophytes associated on *A. macrophylla* led to five new eremophilane sesquiterpenes: xylarenones C–G (**14**–**18**) isolated from solid cultures of *Camarops* sp. (Boliniaceae) [100,101]. This fungus was also able to produce two rearranged sesquiterpenes: 3,5,9-trihydroxy presilphiperfolane (**19**) and 4-deoxy-10-oxodihydrobotrydial (**20**); two branched polyketides: 4-((E)-pent-1-enyl)-3-((1’S,2’S)-1’,2’-dihydroxybut-3-enyl)-5H-furan-2-one (**21**) and (2E,4R)-2,4-dimethylnon-2-enoic acid (**22**); seven phenolic derivatives: *p*-hydroxyphenyllactic acid (**23**), phenyllactic acid (**24**), *p*-hydroxybenzoic acid (**25**), *p*-hydroxybenzaldehyde (**26**), *n*-butyl-3,4- dihydroxybenzoate (**27**), *n*-hexyl-3,4-dihydroxybenzoate (**28**) and *n*-octyl-3,4-dihydroxybenzoate (**29**); and the known compound (2E,4S)-2,4- dimethyloct-2-enoic acid (**30**) (Figure 5) [102].

**Figure 6:**
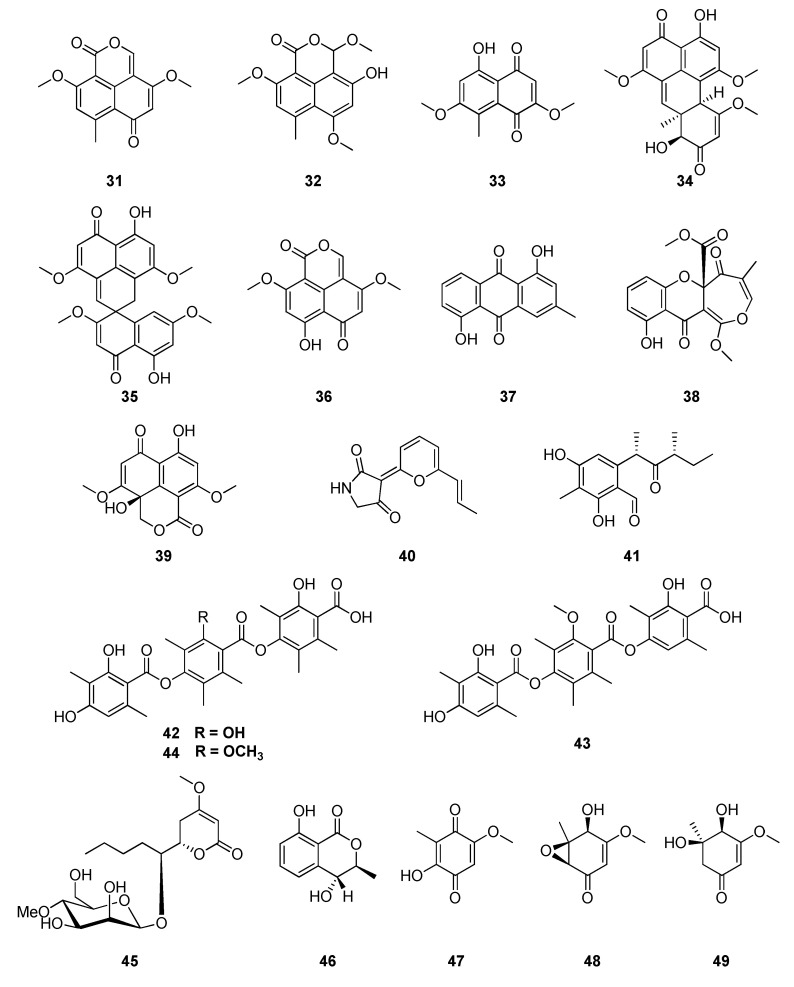
Compounds extracted from endophytic fungus *Sporormiella minimoides* obtained from *Hintonia latiflora.*

Extracts of solid cultures of *Sporormiella minimoides* (Sporormiaceae), isolated as an endophytic fungus from leaves *Hintonia latiflora* collected in Mexico, yielded five polyketides, 3,6-dimethoxy-8-methyl-1H,6Hbenzo[de]isochromene-1,9-dione (**31**), 3-hydroxy-1,6,10-trimethoxy-8-methyl-1H,3H-benzo[de]isochromen-9-one (**32**), 5-hydroxy-2,7-dimethoxy-8-methylnaphthoquinone (**33**), minimoidiones A (**34**) and B (**35**), along with four known compounds: corymbiferone (**36**), ziganein (**37**), brocaenol B (**38**) and preussochromone C (**39**) [103,104,105]. Two other compounds, 9S,11R(+)-ascosalitoxin (**40**) and vermelhotin (**41**), were also produced by endophytes from this plant [105,106]. The tridepsides, secondary metabolites produced by fungus *Chaetomium* sp. (Chaetomiaceae), also isolated from medicinal plant *H. latiflora*, were identified as thielavins A (**42**), J(**43**) and K (**44**) [107]. Two new compounds, pestalotin 4′-O-methyl-β-mannopyranoside (**45**) and 3S,4R-(+)-4-hydroxymellein (**46**), were isolated from an organic extract of *X. feejeensis*, which was isolated from this plant. In addition, the compounds (3S,4S)-4-hydroxymellein (**9**), (3S)-8-methylmellein (**10**), and the quinone derivatives 2-hydroxy-5-methoxy-3-methylcyclohexa-2,5-diene-1,4-dione (**47**), 4S,5S,6S-4-hydroxy-3-methoxy-5-methyl-5,6-epoxycyclohex-2-en-1-one (**48**), and 4R,5R-dihydroxy-3-methoxy-5-methylcyclohexen-2-en-1-one (**49**) were obtained (Figure 6) [108]. 

The ethyl acetate extract from *Cytospora rhizophorae* (Valsaceae), a fungus associated with *Morinda officinalis*, led to the isolation of three new compounds, named cytosporaphenones A–C (**50**–**52**), one new polyhydric benzophenone, and two new naphthopyrone derivatives, respectively. In addition to eight known compounds: 2-(2′S-hydroxypropyl)-5-methyl-7-hydroxychromone (**53**), 2-acetonyl-7-hydroxy-5-methylchromone (**54**), 8-hydroxy-6-methylxanthone-1-carboxylic acid (**55**), regiolone (**56**), (3R,4R)-cis-4-hydroxy-5-methylmellein (**57**), scytalone (**58**), p-hydroxybenzoic acid (**59**) and 4-hydroxy-3-methoxybenzene-ethanol (**60**). Interestingly, all of them were identified from this strain for the first time, and these three new compounds (**50**–**52**) were the most highly oxygenated metabolites of their families discovered in nature [109]. 

**Figure 7:**
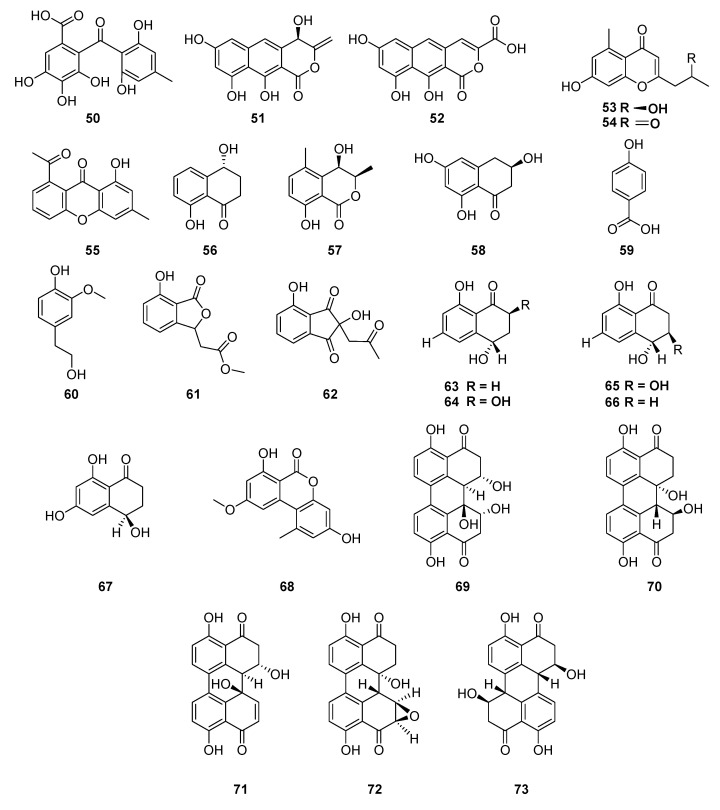
Compounds extracted from endophytic fungal strain *Alternaria* sp. isolated from medicinal plant *M. officinalis.*

The endophytic fungal strain *Alternaria* sp. (Pleosporaceae) isolated from medicinal plant *M. officinalis* produced two new metabolites, isobenzofuranone A (**61**) and indandione B (**62**), together with eleven known compounds (**63**–**73**): isosclerone (**63**), 2,4,8-trihydroxy-1-tetralone (**64**), 3,4-dihydro-3,4,8-trihydroxy-1[2H]-naphthalenone (**65**), 6-hydroxyisosclerone (**66**), *cis*-4-hydroxyscytalone (**67**), alternariol-4-methyl ether (**68**), 6-*epi*-stemphytriol (**69**), dihydroalterperylenol (**70**), alterperylenol (**71**), altertoxin II (**72**) and stemphyperylenol (**73**). It is relevant emphasizing that indandione (**62**) showed a rarely occurring indanone skeleton in natural products (Figure 7) [110].

**Figure 8:**
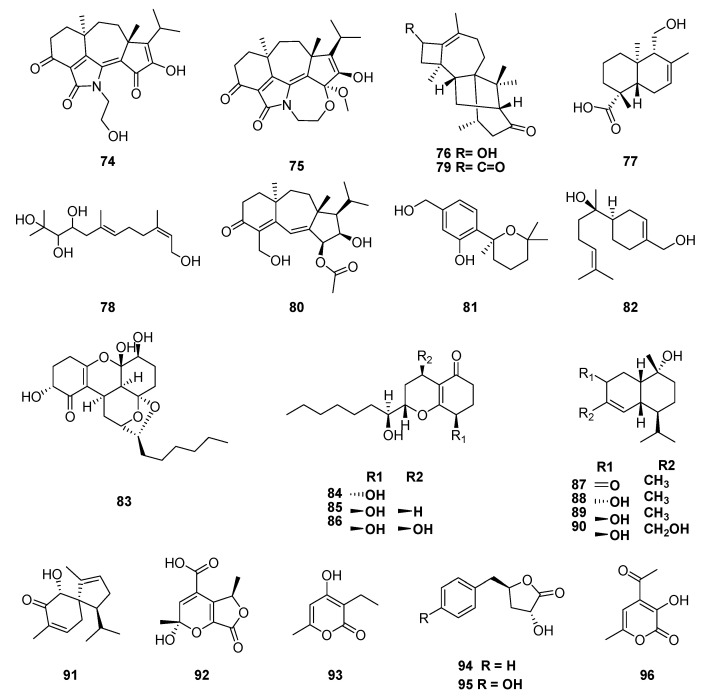
Compounds extracted from endophytic fungus *Trichoderma koningiopsis* (Hypocreaceae), also isolated from *M. officinalis*.

The chemical investigation of the endophytic fungus *Trichoderma koningiopsis* (Hypocreaceae), also isolated from *M. officinalis* yielded three new diterpenes: koninginols A–C (**74**–**76**); two new sesquiterpenoids, 11-hydroxy-15-drimeneoic acid (**77**) and koninginol D (**78**); as well as twelve known metabolites identified as harziandione 2 (**79**), radianspene B (**80**), (S)-(-)-5-(hydroxymethyl)-2-(2′,6′,6′-trimethyltetrahydro-2H-pyran-2-yl)phenol (**81**), hamanasol A (**82**), trichodermatide A (**83**), dihydropyran (**84**), ketodiol (**85**), 7-*O*-methylkoninginin D (**86**), (1S,6R,7S,10R)-10-hydroxy-4(5)-muurolen-3-one (**87**), 1R,3S,6S,7R,10S-7-isopropyl-4,10-dimethylbicyclo[4.4.0]dec-4-en-3,10-diol (**88**), 1R,3R,6S,7R,10S-7-isopropyl-4,10-dimethylbicyclo[4.4.0]dec-4-en-3,10-diol (**89**) and coprinol (**90**) [111]. Recently, six polyketides, 6-hydroxy-4-isopropyl-1,8-dimethylspiro[4.5]deca-1,8-dien-7-one (**91**), 2-hydroxy-2,5-dimethyl-7-oxo-5,7-dihydro-2H-furo[3,4-b]pyran-4-carboxylicacid (**92**), 3- ethyl-4-hydroxy-6-methyl-2H-pyran-2-one (**93**), harzialactone A (**94**), 3-hydroxy-5-(4-hydroxybenzyl)dihydrofuran-2(3H)-one (**95**), and 4-acetyl-3-hydroxy-6-methyl-pyran-2-one (**96**) were isolated from *T. spirale*, another endophytes from *M. officinalis* (Figure 8) [112].

**Figure 9:**
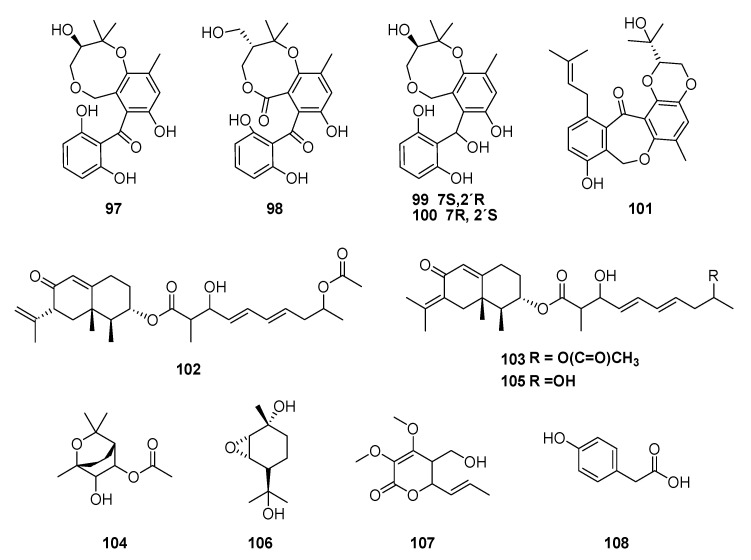
Compounds extracted from endophytic fungi, *Cytospora rhizophorae* and *Diaporthe lithocarpus* obtained from *M. officinalis*.

The endophytic fungi, *Cytospora rhizophorae* and *Diaporthe lithocarpus* (Diaporthaceae), were also obtained from *M. officinalis*. New metabolites isolated from *C. rhizophorae* included cytosporins A–D (**97**–**100**) meroterpenoids. These structures represent the first example of natural products that bear novel benzo[b][1,5]dioxocane framework embodying hemiterpene and benzophenone moieties [113]. Compounds **97**–**100** were evaluated for antimicrobial activities against *Escherichia coli* and *Staphylococcus aureus*. However, the compounds exhibited weak antibiotic activity with inhibition in concentrations above 250 μg mL^−1^. The endophytic fungus, *D. lithocarpus,* yielded tenllone I (**101**), a new benzophenone derivative; two new eremophilane derivatives, lithocarins B (**102**) and C (**103**); a new monoterpenoid, lithocarin D (**104**); tenellone H (**105**); and phomopene (**106**) [114]. Studies of endophytic fungus of Nigerian medicinal plants led to isolation of multiforisin I (**107**) and 4-hydroxyphenylacetic acid (**108**) of *Neurospora discreta* (Sordariaceae) from leaves of *M. lucida* (Figure 9) [115].

**Figure 10:**
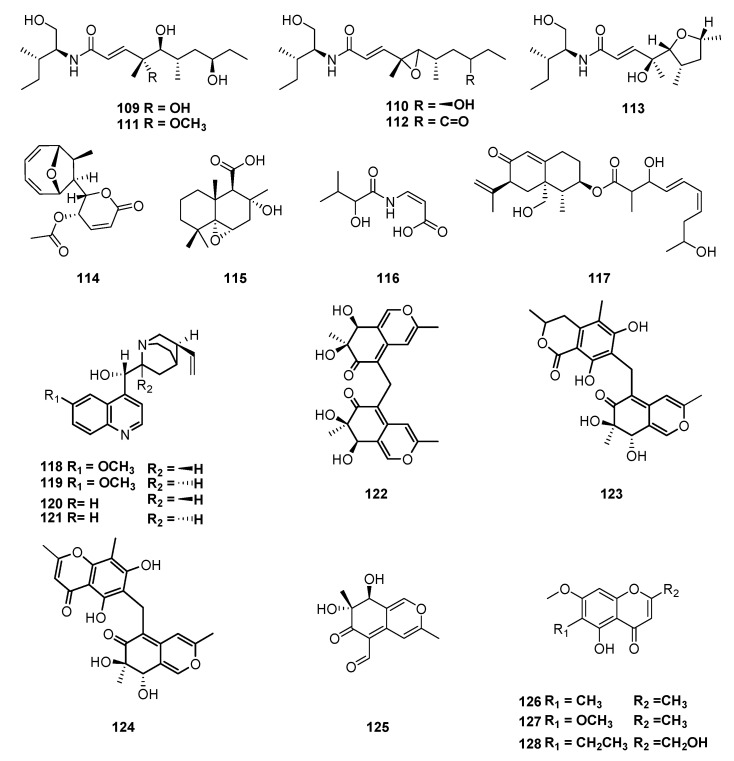
Compounds extracted from endophytic fungi, *Curvularia geniculate, D. pseudomangiferae, Diaporthe, Colletotrichum spp and Mycoleptodiscus indicus obtained from Catunaregam tomentosa, Sabicea cinereal, Cinchona ledgeriana, C. calisaya and Borreria verticillate*, respectively.

The curvularides A–E (**109**–**113**) are hybrid peptide–polyketides isolated from *Curvularia geniculata* (Pleosporaceae), an endophytic fungus obtained from the twigs of *Catunaregam tomentosa*. Their structures contain a 12-carbon atoms polyketide skeleton unit-linked, through an amide bond, with a derivative of L-isoleucine, a rare compound class [116]. The endophytic fungus *D. pseudomangiferae* retrieved from leaves of *Sabicea cinerea* species found along forest edges in the French Guiana, produces four metabolites: mycoepoxydiene (**114**) and altiloxin A (**115**), as well as enamidin (**116**) and eremofortin F (**117**) [117]. A filamentous fungus of the genus *Diaporthe* associated with the seeds of *Cinchona ledgeriana*, from West Java–Indonesia, produces cinchona alkaloids: quinine (**118**), quinidine (**119**), cinchonidine (**120**) and cinchonine (**121**), upon cultivation in a synthetic liquid medium [118,119,120,121,122]. Quinine (**118**), an antimalarial drug, has also been found in chloroform extracts of *Colletotrichum* spp. isolated from *C. calisaya* (Figure 10) [123].

Three new azaphilones with an unusual methylene bridge, named mycoleptones A, B, and C (**122**–**124**), were obtained from cultures of *Mycoleptodiscus indicus* (Magnaporthaceae), a fungus isolated from South American medicinal plant *Borreria verticillate* (Figure 10) [124]. Besides, other polyketides, austidiol (**125**), eugenitin (**126**), 6-methoxieugenin (**127**), and 9-hydroxyeugenin (**128**), were also produced (Figure 10) [124,125].

**Figure 11:**
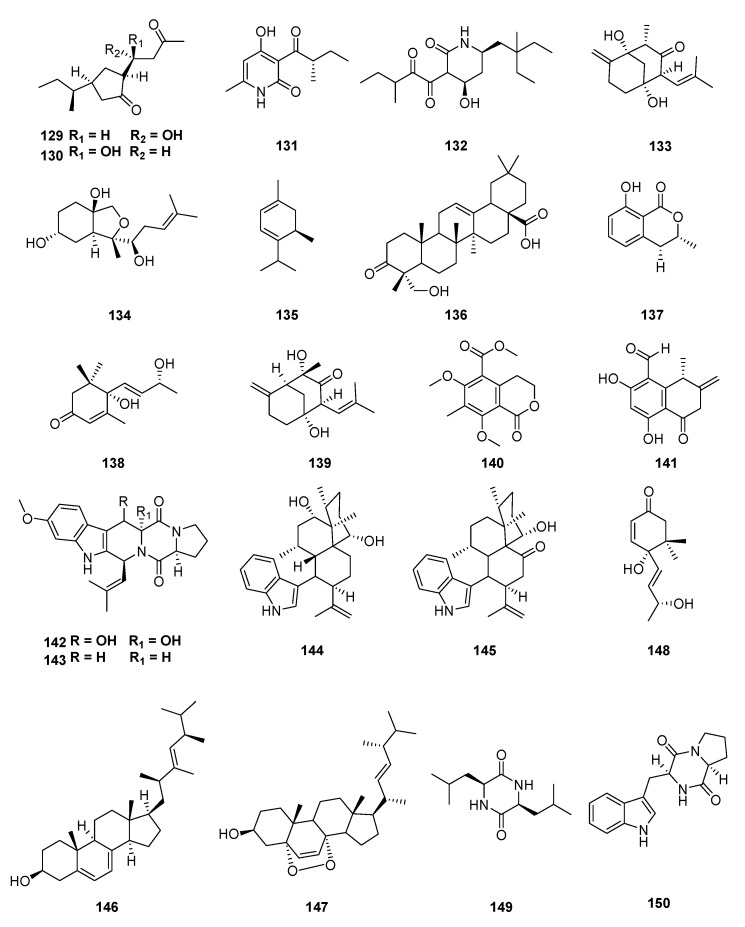
Compounds extracted from endophytic fungi, *C. gloeosporioides* obtained from *Uncaria rhynchophylla.*

A fungus endophyte from *Uncaria rhynchophylla*, *C. gloeosporioides* (Glomerellaceae), produced four novel lactams in culture broth, colletotrilactam A–D (**129**–**132**); colletotrichine A (**133**) and B (**134**); and eleven more compounds: 2-isopropyl-5-methyl-2,4-cyclohexadien-1-ol (**135**), cis-4-hydroxymellein (**9**), 8-methyl-mellein(**10**), hederagonic acid (**136**), mellein (**137**) and blumenol A (**138**), aspergiketone (**139**), djalonenol (**140**), (4S)-(+)-ascochin (**141**), 12,13-dihydroxyfumitremorgin C (**142**) and fumitremorgin C (**143**) [124,125,126]. On the other hand, when grown in wheat bran medium, *C. gloeosporioides* produced nine compounds: 4-epi-14-hydroxy-10, 23-dihydro-24, 25-dehydroaflavinine (**144**), 10, 23-dihydro-24,25-dehydro-21–oxoaflavinine (**145**), ergosterol (**146**), ergosterol peroxide (**147**), mellein (**137**), 4, 5-dihydroblumenol A (**148**), cyclo(L-leucyl-L-leucyl) (**149**), and brevianamide F (**150**). It was the first report of isolation of the compounds **144**, **145**, **148**, **149**, and **150** from the *Colletotrichum* genus (Figure 11) [127].

**Figure 12:**
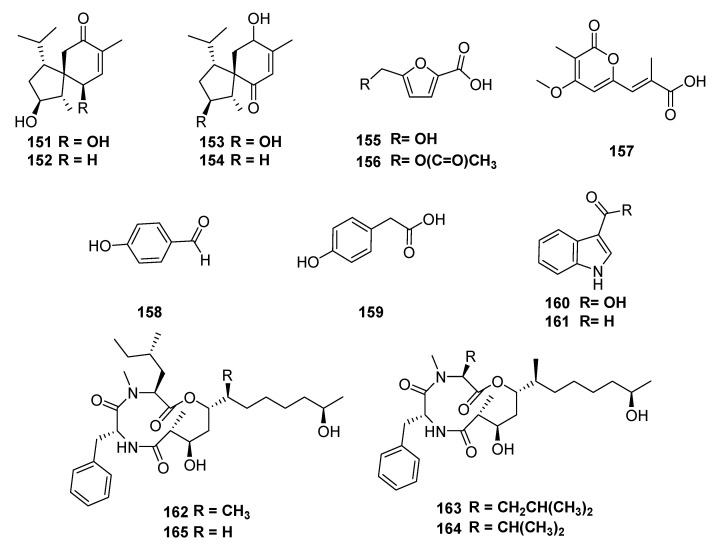
Compounds extracted from endophytic fungi, *Colletricnhum sp.* obtained from *Sabicea cinereal* and *Rubia pondantha*.

However, the chemical investigation of the *C. gloeosporioides* ethyl acetate extract, obtained from a solid culture, isolated from the leaves of *Sabicea cinerea*, led to the isolation of four new acoranes (**151**–**154**) and other seven known compounds: 5-hydroxymethyl-furan-2-carboxylic acid (**155**), 5-acetoxymethyl-furan-2-carboxylic acid (**156**), convolvulopyrone (**157**), p-hydroxybenzaldehyde (**158**), 4-hydroxyphenyl acetic acid (**159**), indole-3-carboxylic acid (**160**) and indole-3-carboxaldehyde (**161**) [128,129]. Recently, four cyclic tridepsipeptides, colletopeptides A−D (**162**–**165**), were isolated from *Colletotrichum* species from stems of *Rubia pondantha*(Figure 12) [130].

*Guignardia* sp. (Botryosphaeriaceae) isolated from the leaves of the mangrove plant *Scyphiphora hydrophyllacea* Gaertn. F., produced six new meroterpenes, guignardones D–I (**166**–**171**); two known compounds, guignardones A (**172**) and B (**173**), and the fatty acid glucoside identified as (R)-3-hydroxyundecanoic acid methylester-3-O-α-L-rhamnopyranoside (**174**) [131,132,133]. Two other antibiotics, brefeldin A (**175**) and trichodermol (**176**), were isolated from endophytic fungus (code C22) from *S. hydrophyllacea* [134].

**Figure 13:**
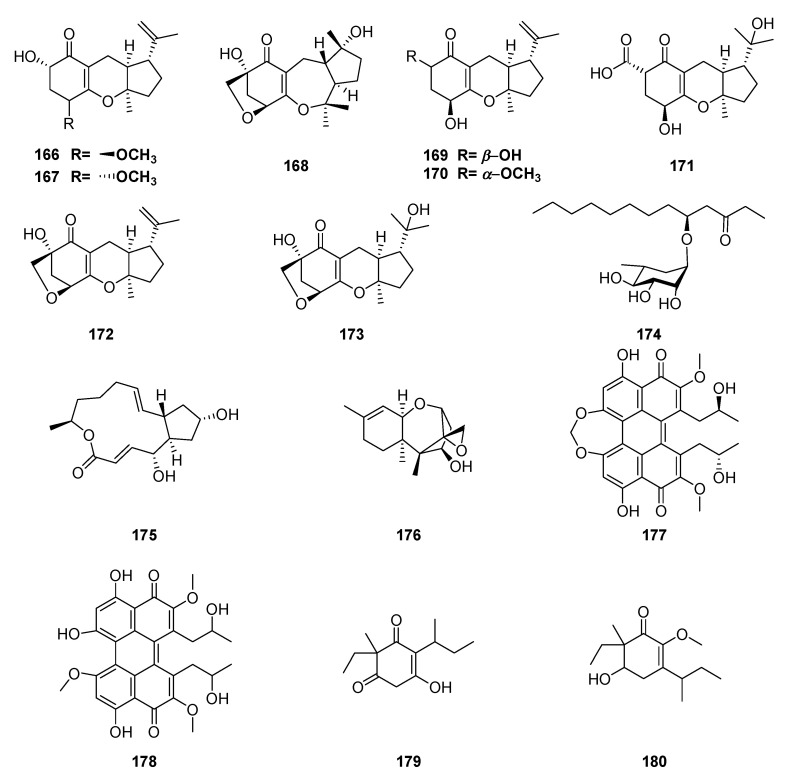
Compounds extracted from endophytic fungi, *Guignardia* sp and *Mycosphaerella* sp. obtained from *Scyphiphora hydrophyllacea* and *Psychotria horizontalis,* respectively.

Analyzing the effect of the culture medium on the production of secondary metabolites by Panamanian endophytic fungi, an antiparasitic compound was obtained, cercosporin (**177**); and a new analog (**178**), isolated from endophytic fungus *Mycosphaerella* sp. (Mycosphaerellaceae), associated with the foliage of *Psychotria horizontalis* [135]. The structures of minor compounds in the extract were elucidated as 2-(2-butyl)-3-hydroxy-6-ethyl-6-methylcyclohex-2-ene-1,5-dione (**179**) and 3-(2-butyl)-6-ethyl-6- methyl-5-hydroxy-2-methoxy-cyclohex-2-eneone (**180**) (Figure 13) [136].

**Figure 14:**
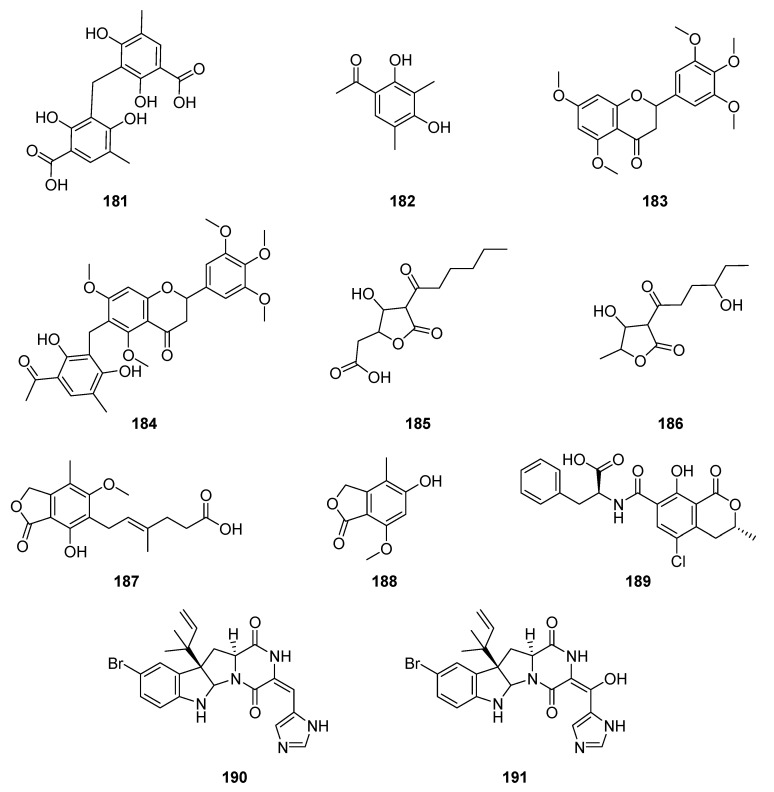
Compounds extracted from endophytic fungi, *P. griseoroseum,* *P. crustosum* and *P. chrysogenum* obtained from *C. arabica.*

In continuous studies on the chemistry of the endophytic fungus *P. griseoroseum* (Trichocomaceae), an endophyte isolated from fruits of *C. arabica*, produced dimethylated tetraketide diclavatol (**181**), clavatol (**182**) and two benzylated flavonoids (**183**–**184**) [137,138]. The studies also resulted in the identification of two known tetronic acids, viridicatic acid (**185**) and terrestric acid (**186**), found in ethyl acetate and n-butanol extract [138]. Mycophenolic acid (**187**), 5-hydroxi-7-methoxy-4-methylphtalide (**188**) and ochratoxin A (**189**) were produced by *P. crustosum* obtained from coffee seeds [139,140]. After adding halides in a broth culture, two bromoroquefortines, 11-bromoroquefortine D (**190**) and 11-bromo-17-hydroxybromoroquefortine C (**191**), were produced by *P. chrysogenum* from leaves of *C. arabica* (Figure 14) [141].

An expedition to Yasuni National Park resulted in the isolation of endophytic *Stelliosphaera formicum* from *Duroia hirsuta*, an understory tree growing in Ecuador. Phylogenetic analysis of this organism describes it as a specimen of a new genus within the order Pleosporales. Besides this organism being an example of new taxonomic diversity, it also produced stelliophaerols A (**192**) and B (**193**), two new sesquiterpene-polyol conjugates 1 [142].

Chemical analyses of *Phomopsis* spp. (Valsaceae) isolated from tropical plants, including *C. arabica,* yielded alternariol (**68**), altenusin (**194**), altenuene (**195**), cytosporones C, O (**196**–**197**), and dothiorelones A–C (**198**–**200**) [143].

**Figure 15:**
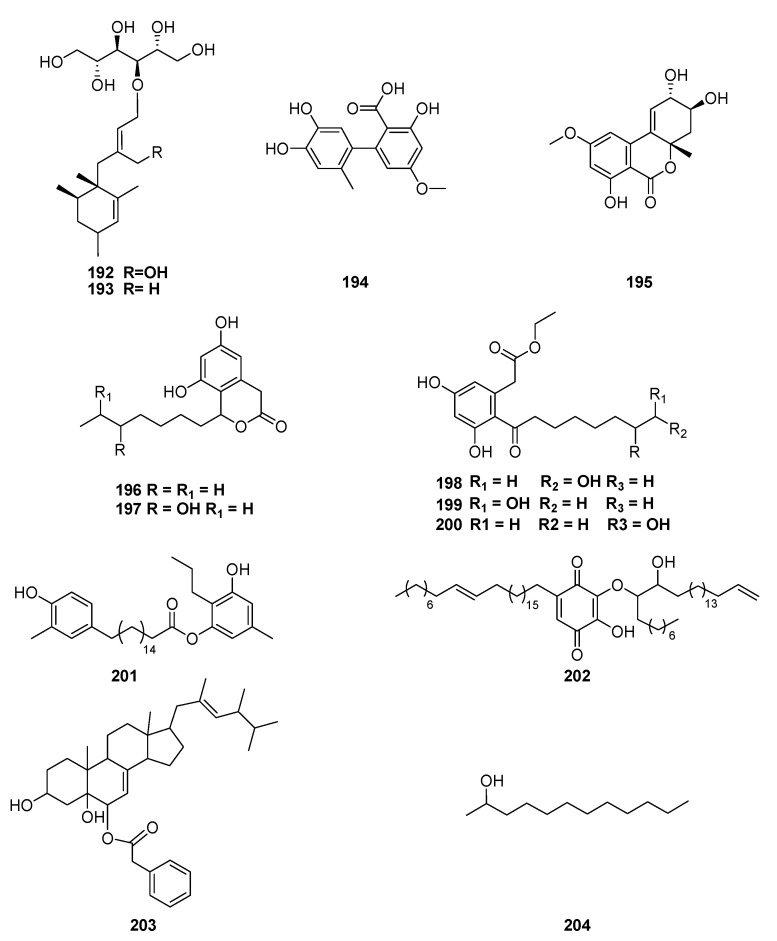
Compounds extracted from endophytic fungi, *Stelliosphaera formicum Phomopsis* spp and *C. cupreum* obtained from *Duroia hirsute,* *C. arabica* and *Mussaenda luteola,* respectively.

Recently, four secondary metabolites from *C. cupreum* associated with *Mussaenda luteola* were characterized as resorcinol (**201**), 6-(heptacosa-18′Z enyl)-2-(18”hydroxyl-1” enyl-19” oxy)-3-hydroxybenzoquinone (**202**), (3β–5α–dihydroxy–6β–phenylacetyloxy–ergosta–7, 22–diene) (**203**) and 2- dodecanol (**204**) (Figure 15) [144,145].

## 3. Biological Activities

Endophyte fungi are capable of synthesizing bioactive compounds, including alkaloids, terpenoids, flavonoids and steroids. Hitherto, most of the secondary metabolites from endophytes are anticancer agents, antibiotics, biological control agents, and other bioactive compounds determined by their different functional roles. In this review, we highlight mainly bioactive natural products endophytically synthetized by endophytic fungi associated with various genera of Rubiaceae (Table 1).

### 3.1. Antifungal and Antibacterial Activity

Thin layer chromatography (TLC) bioautography indicated that compounds **1** and **2** (isolated from *Xylaria* sp.) and compounds **6**, **9**, **10**, **12**, and **13** (isolated from *Penicillium* sp.) display activity against *Cladosporium cladosporioides* and *C. sphaerospermum*. The most active compounds, orcinol (**6**), 4-hydroxymellein (**9**), (R)-7-hydroxymellein (**12**) and (3R,4R)-4,7-dihydroxymellein (**13**) showed a potent effect exhibiting a detection limit of 5.0 and 10.0 μg mL^−1^ against *C. cladosporioides* and *C. sphaerospermum*, respectively [97,98,99]. In a disk diffusion assay, curvularide B (**110**) exhibited activity against *Candida albicans* with an inhibition zone diameter of 12.1 mm; it also showed synergistic effect with a fluconazole drug [116].

Crude extracts of *S. formicum* from *D. hirsuta* showed specific activity against *Staphylococcus aureus*. Stelliosphaerols A (**192**) and B (**193**) were subsequently isolated by bioassay-guided isolation as causative agents of this activity. Following it, the growth inhibition assays revealed minimum inhibitory concentration (MIC) values for stelliosphaerols A and B of approximately 250 μg mL^−1^ [142]. On the other hand, the meroterpene guignardone I (**171**), guignardone B (**173**) and the fatty acid glucoside (**174**) produced by the endophytic fungal from *S. hydrophyllacea* showed modest inhibitory effects on *S. aureus* and methicillin-resistant *S. aureus* (MRSA) [132,133].

Two new diterpenes, koninginols A (**74**) and B (**75**), isolated from the endophytic fungus *T. koningiopsis* derived from *M. officinalis*, exhibited significant antibacterial activity against *B. subtilis* with MIC values of 10 and 2 μg mL^−1^, respectively [111]. Moreover, the alkaloid 11-bromoroquefortine D (**190**) was also able to inhibit this bacterium at a concentration of 15 mM [141]. Metabolites **201**, **202** and **203** isolated from *C. cupreum* showed anti-mycobacterial activity against *Mycobacterium tuberculosis*, with MIC values of 6.3, 6.25 and 25 μg mL^−1^ [145].

### 3.2. Neurodegenerative Diseases

Acetylcholinesterase (AChE) and monoamine oxidase (MAO) are enzymatic targets for the search of new drugs for the treatment of neurodegenerative diseases [147,148,149,150]. Diketopiperazine **7** and the dihydroisocoumarins **12**, **13**, and **9**, isolated from *Penicillium* sp. associated with *A. macrophylla*, exhibited AChE inhibitory activity and showed a detection limit of 10.0 μg (**7**, **12**, **13**) and 30.0 μg (**9**), respectively [98,99]. On the other hand, xylarenone C (**14**) isolated from *Camarops* sp., had a minimum AChE inhibitory concentration of 6.25 μg, while the others compound (**15**, **22**, and **30**) from *Camarops* sp., showed weak acetylcholinesterase (AChE) inhibitory activity [101]. Recently, the sesquiterpenoids colletotrichines A(**133**) and B (**134**) produced by *C. gloeosporioides* inhibited AChE activity with the half-maximal inhibitory concentration IC_50_ values of 28.0 and 38.0 μg mL^−1^, respectively [125,126].

Monoamine oxidase (MAO) is an enzyme that catalyzes the oxidative deamination of biogenic amines neurotransmitters. Besides, MAO plays an essential role in the central nervous system and peripheral organs [151,152]. Compound mellein (**137**), produced by *C. gloeosporioides*, showed potent MAO inhibitory activity with an IC_50_ value of 8.93 ± 0.34 μg mL^−1^, while the standard, iproniazid, was 1.80 ± 0.5 μg mL^−1^ [124].

### 3.3. Cytotoxic Activity

The eremophilane sesquiterpenes xylarenone C (**14**) and xylarenone D (**15**), isolated from *Camarops* sp., exhibited cytotoxic activity against human tumor cell lines, such as leukemia (HL-60), melanoma (MDA/MB-435), colon (HCT-8), and glioblastoma (SF-295). The antiproliferative effect was evaluated following 72h of treatment, and the compounds **14** and **15** were more active against MDA/MB-435 (IC_50_ = 2.4 μg mL^−1^) and HL-60 (IC_50_ = 1.2 μg mL^−1^) cells, respectively [101]. Eremophilane sesquiterpene compounds exhibit phytotoxic potential; antifungal, antibacterial, carcinostatic, and cytotoxic activities; and can act as a phytohormone [100,153].

Cytosporaphenone A (**50**), produced by the fungus *C. rhizophorae,* which is derived from *M. officinalis,* showed uncommon antiproliferative activity against MCF-7 and HepG-2 cell lines at a concentration of 100 μg mL^−1^, with inhibition rates of 91.0% and 80.5%, respectively [109]. The compounds **70** and **71** from the filamentous fungus *Alternaria* sp. reduced the viability of four human tumor cells lines: MCF-7, HepG-2, NCI-H460, and SF-268 with IC_50_ values ranging from 1.91 to 9.67 μM [110]. In addition, the multiforisin I (**107**) produced by *N. discreta* showed moderate activity against lymphoma cells, reducing 70% cell growth [115].

Bioassay-guided fractionation of ethyl acetate extract from *D. pseudomangifera*, by cytotoxic effects against mammalian cancer cells allowed the isolation of the mycoepoxydien (**114**), which showed cytotoxic activity with IC_50_ values of 7.5, 17.7, and 15.8 μM against against human uterine cervical carcinoma KB and MDA-MB-435 cells, respectively. The compound eremofortin F (**117**) was cytotoxic on KB (IC_50_ = 13.9 μM) and MRC5 (IC_50_ = 12.2 μM) cells [117]. The azaphilone mycoleptone B (**122**) isolated from *M. indicus* associated with *B. verticillata* presented cytotoxic activity against human prostate cancer (PC3) cells (IC_50_ = 7.1 ± 3.8 μM). However, when compared with doxorubicin, the reference compound for cytotoxicity assays, its activity was lower [146]. Cercosporin (**177**), produced by *Mycosphaerella* sp. associated with *P. horizontalis,* showed lower cytotoxicity to mammalian Vero cells (1.54 μM) and high potency against MCF7 cancer cell lines (IC_50_ = 4.68 μM). The analog compound (**178**) was not active in these assays [136].

The compounds isolated from the endophytic fungus *T. koningiopsis* were evaluated for cytotoxic activity against HepG-2, MCF-7, SF-268 and A549 cells lines. The compounds koninginol B (**75**), 1R,3S,6S,7R,10S-7-isopropyl-4,10-dimethylbicyclo[4.4.0]dec-4-en-3,10-diol (**88**) and 1R,3R,6S,7R,10S-7-isopropyl-4,10-dimethylbicyclo[4.4.0]dec-4-en-3,10-diol (**89**) showed antiproliferative activities against A549 with IC_50_ values of 46.6, 31.3 and 22.2 μM, respectively [111]. However, none of the metabolites isolated from *T. spirale* (**91**–**96**) presented cytotoxicity activity against cancer cell lines [112].

The compounds from *D. lithocarpus*, another endophyte also isolated from *M. officinalis*, were tested for their cytotoxic activity by the sulforhodamine B method on four human tumor cell lines (SF-268, MCF-7, HepG-2 and A549). The compounds lithocarin B (**102**), lithocarin C (**103**), and tenellone H (**105**) presented IC_50_ values ranging from 30 to 100 μM in the four tumor cell lines selected [114].

Cancer human cell proliferation (SF-295 and HTC-116) was inhibited by 11-bromo-roquefortine D (**190**) with rates of 63% and 6.7%, at a concentration of 5.3 μM, respectively [141]. The cytotoxicity activity of 6-(heptacosa-18′Zenyl)-2-(18”hydroxyl-1”enyl-19” oxy)-3-hydroxybenzoquinone (**202**) and (3β–5α–dihydroxy–6β–phenylacetyloxy–ergosta–7, 22–diene) (**203**) was evaluated against a breast cancer cell line (MCF-7). They reduced the cell viability by 52% and 49%, respectively, at a concentration of 100μg.mL^−^^1^ [145].

The PI3Kα inhibitory activity of compounds isolated from *C. gloeosporioides*, an endophytic fungus from *U. rhynchophylla,* was evaluated. The phosphoinositide 3-kinases (PI3Ks), a family of lipid kinases, showed a crucial regulatory role in many cellular processes, including cell proliferation, especially PI3Kα as one of the main targets for therapeutic intervention in cancer [154]. Hence, compounds from *C. gloeosporioides* were tested for their phosphoinositide 3-kinase (PI3Kα) inhibitory activity. The compounds cyclo(L-leucyl-l-leucyl) (**149**) and brevianamide F (**150**) showed potent PI3Kα inhibitory activity with IC_50_ values of 38.1 and 4.8 μM, respectively, while the other compounds showed weak activity at a concentration of 20 μg·mL^−1^ [127].

### 3.4. Anti-Inflammatory and Antioxidant Activity

Xylarenones C, D, F, and G (**14**, **15**, **17**, **18**) obtained from broth cultures by *Camarops* sp. showed meaningful inhibitory effect of reactive oxygen species (ROS) produced by stimulated neutrophils. The inhibitory concentrations of **14** (IC_50_ = 6.13 ± 0.41 μM), **15** (IC_50_ = 5.73 ± 0.42 μM), **17** (IC_50_ = 5.90 ± 0.70 μM), and **18** (IC_50_ = 4.17 ± 0.81 μM) were similar to those of quercetin and apocynin, an efficient inhibitor of the NADPH (nicotinamide adenine dinucleotide phosphate) oxidase complex. Furthermore, the compounds **14**, **15**, **17** and **18** were also evaluated for their radical scavenging properties in different analytical methods, such as scavengers of superoxide anions (the first ROS produced via the NADPH oxidase complex by stimulated neutrophils), HOCl (the main strong oxidant produced by myeloperoxidase (MPO)), and MPO enzymatic activity, however, the compounds were inactive and had IC_50_ values of >100 μM [100].

Colletopeptide A (**162**) isolated from *Colletotrichum* sp. showed significant anti-inflammatory activity; it inhibited the effects of lipopolysaccharide-induced nitric oxide production with an IC_50_ value of 8.3 μM. The other colletopeptides, B (**163**), C (**164**) and D (**165**), also inhibited the lipopolysaccharide (LPS)-induced nitric oxide production, with IC_50_ values of 38.7, 13.5 and 22.2 μM, respectively [130].

### 3.5. Anti-Protozoal Activity

Azaphilones mycoleptones A and B (**122**–**123**) and the polyketide austidiol (**125**) isolated from *M. indicus* presented in vitro leishmanicidal activity, being active against *Leishmania donovani,* with IC_50_ values of 28.5, 21.7 and 20.5 μM, respectively [146].

The in vitro assay results suggest that cercosporin (**177**) is highly active against *Plasmodium falciparum* (IC_50_ = 1.03 μM), *L. donovani* (IC_50_ = 0.46 μM), and *Trypanosoma cruzi* (IC_50_ = 1.08 μM). Nevertheless, the bioactivity profile observed for cercosporin indicated that it was not specific for any the assayed parasites [127,128]. Compound **178**, identified as a seven-membered dioxepane ring-opened analogue of cercosporin, showed a significant reduction in activity in all these biological assays (IC_50_ >10 μg mL^−1^), indicating the importance of the methylenedioxy functionality to the biological properties of compound **177** [136]. On the other hand, the alkaloid quinine (**118**) produced by *Diaporthe* sp. is a well-known antimalarial drug that is effective against the erythrocyte stage of the parasite *P. falciparum* [120,121,146].

### 3.6. Hyperglycemic Control

The best treatment for type 2 diabetes mellitus (TII-DM) involves hyperglycemic control using appropriate therapies. In recent years, substantial efforts to discover effective inhibitors of α-glucosidases from natural sources have been made [103,104]. The polyketide mimimoidione A (**34**) isolated from *S. minimoides* showed an excellent activity against *Saccharomyces cerevisiae* α-glucosidase (α-GHY), with an IC_50_ of 2.9 μM [104]. One the other hand, the tridepsides thielavins A (**42**), J (**43**) and K (**44**), isolated from *Chaetomium* sp. from *H. latiflora*, inhibited the α-GHY with IC_50_ values of 23.8, 15.8, and 22.1 μM, respectively. Their inhibitory action was better than the acarbose standard (IC_50_ = 545 μM). Thielavin J (**43**) inhibited the activity of α-glucosidase from *B. stearothermophilus* (αGHBs) with an IC_50_ = 30.5 μM, being less active than acarbose (IC_50_ = 0.015 μM) [107]. The thielavin K (**44**) reduced fasting and postprandial glucose levels in a TII-DM animal model. Therefore, thielavin-type tridepsides represent a new class of α-glucosidase inhibitors and can become hypoglycemic agents for the treatment of TII-DM [107].

The compounds 3S,4R-(+)-4-hydroxymellein (**46**) and 3S,4S-(+)-4-hydroxymellein (**9**) inhibited the activity of enzyme *S. cerevisiae* α-glucosidase, with IC_50_ values of 441 ± 23 and 549 ± 2.5 μM, respectively [108]. Six compounds from *Alternaria* sp., namely, 2,4,8-trihydroxy-1-tetralone (**64**), 3,4-dihydro-3,4,8-trihydroxy-1[2H]-naphthalenone (**65**), 6-epi-stemphytriol (**69**), dihydroalterperylenol (**70**), altertoxin II (**72**) and stemphyperylenol (**73**), demonstrated prominent inhibitory activities against α-glucosidase (αGHY). The IC_50_ values in the range of 12.05 to 166.13 μM, observed for these compounds, were clearly better and more significant when compared to the positive control of acarbose (IC_50_ = 427.34 μM) [110].

### 3.7. Other Activities

Proteases are relevant enzymatic targets because these proteins control the formation of functional peptides that participate in physiological processes [155]. The protease inhibitory activity of compounds xylarenones C–E (**14**–**16**) was evaluated in vitro using the enzymes subtilisin and pepsin. A potent inhibitory activity for the pepsin and subtilisin in protease assays was observed for compound **14** with an IC_50_ of 0.288 and 0.462 μM, respectively. However, compounds **15** and **16** displayed no inhibitory activity on subtilisin (<10%) at any of the four concentrations tested (1.00, 0.1, 0.01, and 0.001 μM) [153].

Metabolites produced by *S. minimoides* were evaluated as potential human calmodulin (hCaM) inhibitors, and two compounds, 5-hydroxy-2,7-dimethoxy-8-methylnaphthoquinone (**33**) and vermelhotin (**41),** quenched the extrinsic fluorescence of this biosensor significantly, with dissociation constant (Kd) values of 1.55 μM and 0.25 μM, respectively. The docking displayed studies to predict the interaction of **33** with hCaM and many hydrophobic interactions with Phe19, Phe68, Met51, Met71, Met72 and Ile52. However, vermelhotin (**41**) showed hydrophobic interactions with Phe92, Met109, Met124, Glu127, Ala128, and Met144 [103,106].

## 4. Conclusions

As demonstrated in this paper, an increasing number of publications revealed a significant interest in endophytes from the Rubiaceae family in recent years due to pharmacological activities. This review presents the chemical diversity and pharmacological properties of secondary metabolites produced by endophyte fungi associated with various genera of Rubiaceae. Several classes of natural products are described for this endophyte, although this study highlights the importance of some metabolites which are involved in antifungal, antibacterial, and anti-protozoal activities; neurodegenerative diseases, cytotoxic activity, anti-inflammatory and antioxidant activity; and hyperglycemic control.

## Figures and Tables

**Table 1:** Compounds produced by endophytic fungi associated with various genera of Rubiaceae and their respectively biological activities.

Classification	Compound	Endophytic	Species	Biological Activities	Refrence
alkaloid	cytochalasin D (**2**)	*Xylaria* sp.	*Palicourea marcgravii*	antifungal	[97]
alkaloid	quinine (**118**)	*Colletotrichum* spp.	*Cinchona ledgeriana*	antiprotozoal	[120,121,146]
alkaloid	brevianamide F (**150**)	*Colletotrichum gloeosporioides*	*Uncaria rhynchophylla*	cytotoxic	[127]
alkaloid	11-bromoroquefortine (**190**)	*Penicillium chrysogenum*	*Coffea arabica*	antibacterial, antiprotozoal, cytotoxic	[141]
coumarin	4-hydroxy-mellein (**9**)	*Penicillium* sp.	*Alibertia macrophylla*	antifungal, acetylcholinesterase inhibitor, anti-hyperglycemic	[98]
coumarin	8-methyl-mellein (**10**)	*Penicillium* sp.	*Alibertia macrophylla*	Antifungal	[98]
coumarin	(R)-7-hydroxymellein (**12**)	*Penicillium* sp.	*Alibertia macrophylla*	antifungal, acetylcholinesterase inhibitor	[99]
coumarin	(3R,4R)-4,7-dihydroxymellein (**13**)	*Penicillium* sp.	*Alibertia macrophylla*	antifungal, acetylcholinesterase inhibitor	[99]
coumarin	3S,4R-(+)-4-hydroxymellein (**46**)	*Xylaria feejeensis*	*Hintonia latiflora*	anti-hyperglycemic	[108]
coumarin	mellein (**137**)	*Colletotrichum gloeosporioides*	*Uncaria rhynchophylla*	monoamine oxidase inhibitor	[127]
diketopiperazine	cyclo-(L-Pro-L-Val) (**7**)	*Penicillium* sp.	*Alibertia macrophylla*	acetylcholinesterase inhibitor	[98]
diketopiperazine	cyclo(L-Leu-L-Leu) (**149**)	*Colletotrichum gloeosporioides*	*Uncaria rhynchophylla*	cytotoxic	[127]
diterpene	koninginol A (**74**)	*Trichoderma koningiopsis*	*Morinda officinalis*	antifungal	[111]
diterpene	koninginol B (**75**)	*Trichoderma koningiopsis*	*Morinda officinalis*	antifungal, cytotoxic	[111]
fatty acid	(R)-3-hydroxyundecanoic acid methylester-3-O-α-L-rhamnopyranoside (**174**)	*Guignardia* sp.	*Scyphiphora hydrophyllacea*	antibacterial	[133]
meroterpene	guignardone I (**171**)	*Guignardia* sp.	*Scyphiphora hydrophyllacea*	antibacterial	[132]
meroterpene	guignardone B (**173**)	*Guignardia* sp.	*Scyphiphora hydrophyllacea*	antibacterial	[132]
phenolic compound	orcinol (**6**)	*Penicillium* sp.	*Alibertia macrophylla*	antifungal	[98]
phenolic compound	thielavins A (**42**)	*Chaetomium* sp.	*Hintonia latiflora*	anti-hyperglycemic	[107]
phenolic compound	thielavins J (**43**)	*Chaetomium* sp.	*Hintonia latiflora*	anti-hyperglycemic	[107]
phenolic compound	thielavins K (**44**)	*Chaetomium* sp.	*Hintonia latiflora*	anti-hyperglycemic	[107]
phenolic compound	cytosporaphenone A (**50**)	*Cytospora rhizophorae*	*Morinda officinalis*	cytotoxic	[109]
phenolic compound	resorcinol (**201**)	*Chaetomium cupreum*	*Mussaenda luteola*	antibacterial	[145]
polyketide	2-hexyl-3-methyl-butanodioic acid (**1**)	*Xylaria* sp.	*Palicourea marcgravii*	antifungal	[97]
polyketide	(2E,4R)-2,4-dimethylnon-2-enoic acid (**22**)	*Camarops* sp.	*Alibertia macrophylla*	acetylcholinesterase inhibitor	[102]
polyketide	(2E,4S)-2,4-dimethyloct-2-enoic acid (**30**).	*Camarops* sp.	*Alibertia macrophylla*	acetylcholinesterase inhibitor	[102]
polyketide	5-hydroxy-2,7-dimethoxy-8-methylnaphthoquinone (**33**)	*Sporormiella minimoides*	*Hintonia latiflora*	human calmodulin inhibitor	[103]
polyketide	minimoidione (**34**)	*Sporormiella minimoides*	*Hintonia latiflora*	anti-hyperglycemic	[104]
polyketide	vermelhotin (**41**)	*Sporormiella minimoides*	*Hintonia latiflora*	human calmodulin inhibitor	[106]
polyketide	2,4,8-trihydroxy-1-tetralone (**64**)	*Alternaria* sp.	*Morinda officinalis*	anti-hyperglycemic	[110]
polyketide	3,4-dihydro-3,4,8-trihydroxy-1[2H]-naphthalenone (**65**)	*Alternaria* sp.	*Morinda officinalis*	anti-hyperglycemic	[110]
polyketide	6-hydroxy-4-isopropyl-1,8-dimethylspiro[4.5]deca-1,8-dien-7-one (**91**)	*Trichoderma spirale*	*Morinda officinalis*	cytotoxic	[112]
polyketide	2-hydroxy-2,5-dimethyl-7-oxo-5,7-dihydro-2H-furo[3,4-b]pyran-4-carboxylicacid (**92**)	*Trichoderma spirale*	*Morinda officinalis*	cytotoxic	[112]
polyketide	3-ethyl-4-hydroxy-6-methyl-2H-pyran-2-one (**93**)	*Trichoderma spirale*	*Morinda officinalis*	cytotoxic	[112]
polyketide	harzialactone A (**94**)	*Trichoderma spirale*	*Morinda officinalis*	cytotoxic	[112]
polyketide	3-hydroxy-5-(4-hydroxybenzyl)dihydrofuran-2(3H)-one (**95**)	*Trichoderma spirale*	*Morinda officinalis*	cytotoxic	[112]
polyketide	4-acetyl-3-hydroxy-6-methyl-pyran-2-one (**96**)	*Trichoderma spirale*	*Morinda officinalis*	cytotoxic	[112]
polyketide	multiforisin I (**107**)	*Neurospora discrete*	*Morinda lucida*	cytotoxic	[115]
polyketide	curvularide B (**110**)	*Curvularia geniculata*	*Catunaregam tomentosa*	antifungal	[116]
polyketide	mycoepoxydiene (**114**)	*Diaporthe pseudomangiferae*	*Cinchona ledgeriana*	cytotoxic	[117]
polyketide	mycoleptones A (**122**)	*Mycoleptodiscus indicus*	*Borreria verticillata*	antiprotozoal, cytotoxic	[146]
polyketide	mycoleptones B (**123**)	*Mycoleptodiscus indicus*	*Borreria verticillata*	antiprotozoal	[130]
polyketide	austidiol (**125**)	*Mycoleptodiscus indicus*	*Borreria verticillata*	antiprotozoal	[130]
polyketide	colletopeptide A (**162**)	*Colletotrichum* sp.	*Rubia pondantha*	anti-inflammatory, antioxidant	[130]
polyketide	colletopeptide B (**163**)	*Colletotrichum* sp.	*Rubia pondantha*	anti-inflammatory, antioxidant	[130]
polyketide	colletopeptide C (**164**)	*Colletotrichum* sp.	*Rubia pondantha*	anti-inflammatory, antioxidant	[130]
polyketide	colletopeptide D (**165**)	*Colletotrichum* sp.	*Rubia pondantha*	anti-inflammatory, antioxidant	[130]
quinone	6-*epi*-stemphytriol (**69**)	*Alternaria* sp.	*Morinda officinalis*	anti-hyperglycemic	[110]
quinone	dihydroalterperylenol (**70**)	*Alternaria* sp.	*Morinda officinalis*	anti-hyperglycemic, cytotoxic	[110]
quinone	alterperylenol (**71**)	*Alternaria* sp.	*Morinda officinalis*	cytotoxic	[110]
quinone	altertoxin II (**72**)	*Alternaria* sp.	*Morinda officinalis*	anti-hyperglycemic	[110]
quinone	stemphyperylenol (**73**)	*Alternaria* sp.	*Morinda officinalis*	antifungal, anti-hyperglycemic	[110]
quinone	cercosporin (**177**)	*Mycosphaerella* sp.	*Psychotria horizontalis*	antiprotozoal, cytotoxic	[127,128,135]
quinone	6-(heptacosa-18′Zenyl)-2-(18”hydroxyl-1”enyl-19” oxy)-3-hydroxybenzoquinone (**202**)	*Chaetomium cupreum*	*Mussaenda luteola*	antibacterial, cytotoxic	[145]
sesquiterpene	xylarenones G (**18**)	*Camarops* sp.	*Alibertia macrophylla*	anti-inflammatory	[100]
sesquiterpene	1R,3S,6S,7R,10S-7-isopropyl-4,10-dimethylbicyclo[4.4.0]dec-4-en-3,10-diol (**88**)	*Trichoderma koningiopsis*	*Morinda officinalis*	cytotoxic	[111]
sesquiterpene	1R,3R,6S,7R,10S-7-isopropyl-4,10-dimethylbicyclo[4.4.0]dec-4-en-3,10-diol (**89**)	*Trichoderma koningiopsis*	*Morinda officinalis*	cytotoxic	[111]
sesquiterpene	lithocarin B (**102**)	*Diaporthe lithocarpus*	*Morinda officinalis*	cytotoxic	[114]
sesquiterpene	lithocarin C (**103**)	*Diaporthe lithocarpus*	*Morinda officinalis*	cytotoxic	[114]
sesquiterpene	tenellone H (**105**)	*Diaporthe lithocarpus*	*Morinda officinalis*	cytotoxic	[114]
sesquiterpene	eremofortin F (**117**)	*Diaporthe pseudomangiferae*	*Cinchona ledgeriana*	cytotoxic	[117]
sesquiterpene	colletotrichine A (**133**)	*Colletotrichum gloeosporioides*	*Uncaria rhynchophylla*	acetylcholinesterase inhibitor	[125]
sesquiterpene	colletotrichine B (**134**)	*Colletotrichum gloeosporioides*	*Uncaria rhynchophylla*	acetylcholinesterase inhibitor	[126]
sesquiterpene	stelliophaerols A (**192**)	*Stelliosphaera formicum*	*Duroia hirsuta*	antibacterial	[142]
sesquiterpene	stelliophaerols B (**193**)	*Stelliosphaera formicum*	*Duroia hirsuta*	antibacterial	[142]
steroid	(3β–5α–dihydroxy–6β–phenylacetyloxy–ergosta–7, 22–diene) (**203**)	*Chaetomium cupreum*	*Mussaenda luteola*	antibacterial, cytotoxic	[145]
